# A stage-specific cell-manipulation platform for inducing endothelialization on demand

**DOI:** 10.1093/nsr/nwz188

**Published:** 2019-11-21

**Authors:** Qilong Zhao, Juan Wang, Yunlong Wang, Huanqing Cui, Xuemin Du

**Affiliations:** Institute of Biomedical & Health Engineering, Shenzhen Institutes of Advanced Technology, Chinese Academy of Sciences, Shenzhen 518035, China

**Keywords:** biomaterials, tissue engineering, shape-memory polymer, topographical conversion, endothelialization

## Abstract

Endothelialization is of great significance for vascular remodeling, as well as for the success of implanted vascular grafts/stents in cardiovascular disease treatment. However, desirable endothelialization on synthetic biomaterials remains greatly challenging owing to extreme difficulty in offering dynamic guidance on endothelial cell (EC) functions resembling the native extracellular matrix-mediated effects. Here, we demonstrate a bilayer platform with near-infrared-triggered transformable topographies, which can alter the geometries and functions of human ECs by tunable topographical cues in a remote-controlled manner, yet cause no damage to the cell viability. The migration and the adhesion/spreading of human ECs are respectively promoted by the temporary anisotropic and permanent isotropic topographies of the platform in turn, which appropriately meet the requirements of stage-specific EC manipulation for endothelialization. In addition to the potential of promoting the development of a new generation of vascular grafts/stents enabling rapid endothelialization, this stage-specific cell-manipulation platform also holds promise in various biomedical fields, since the needs for stepwise control over different cell functions are common in wound healing and various tissue-regeneration processes.

## INTRODUCTION

In the human body, tissue morphogenesis and regeneration are usually complex processes, involving the dynamic regulation of multiple cell functions by endogenous and exogenous signals at different stages [1,2]. Endothelialization, with great significance in vascular remodeling, is a typical example that requires dynamic guidance on endothelial cell (EC) functions by the native extracellular matrix (ECM) [3]. Within the native endothelialization process, the recruitment/migration and the adhesion/spreading of ECs are promoted sequentially at the early and the later stages, respectively, to form a confluent EC monolayer, guided by different ECM-mediated effects [3]. Since undesirable endothelialization is the major cause of vascular disorders and the failure of implanted vascular grafts/stents, numerous efforts have been made for promoting endothelialization. Despite some success in engineering biomimetic microenvironments in supporting the growth and self-organization of ECs for endothelialization, existing strategies, such as either constructing tissue-engineering scaffolds with similar compositions or bioactivities to the native ECM of the endothelium [4,5] or building EC-laden biohybrid constructs with similar architecture to the anatomy of vessels through microfluidic-based bioprinting methods [6–8], on-demand cell manipulation at different stages of endothelialization resembling the native ECM-mediated effects still cannot be achieved.

To replicate the dynamic ECM-mediated effects, artificial cell microenvironments capable of delivering changing inducements [9–12] or offering dynamic stimulus [13–15] have been emerging. Notably, topographical arrangement as an inducing cue of biomaterials is particularly effective in directing cell behaviors, which affects cell geometries and subsequently a cascade of mechanotransduction signaling [16–19], even stem-cell fate [20,21]. The control over cell anisotropy by biomaterial topography has shown multiple potentials for the repair of different human tissues such as tendon/ligament and nerve [22,23], as well as for directing the geometries and functions of ECs [24]. However, biomaterials with static topographies are unlikely to offer stage-specific guidance on EC functions like the native ECM throughout the endothelialization process. Recently, some actuating biomaterials with programmable reconfigurations have been developed based on responsive hydrogels and shape-memory polymers [25–27], which provide promising candidates for constructing artificial cell microenvironments with dynamic properties, particularly transformable topographies, to offer changing inducements for cell manipulation [28–34]. However, since desirable endothelialization requires elaborately regulating different EC functions in a well-defined sequential manner, developing actuating biomaterials with precisely controlled features capable of effectively switching the behaviors and functions of ECs to appropriately meet the complicate cell-manipulation requirements throughout the endothelialization process remains challenging.

To address the unmet need of offering on-demand guidance on EC behaviors and functions in a well-defined spatiotemporal manner, we establish a bilayer platform with remote-controlled transformable topographies to induce on demand different EC functions for improving endothelialization. Unlike most of existing actuating biomaterials whose topographical alterations rely on the change of overall environmental temperature, our bilayer platform consisting of one photothermal layer and one shape-memory layer can both durably preserve its temporary topography and readily convert its topographies at the unaltered physiological temperature (37°C). Importantly, a light at the near-infrared (NIR) region (808 nm) with deep tissue penetration can remotely trigger the topographical conversion of the platform. Through NIR-controlled topographical conversion of the bilayer platform from a temporary microgroove array to a permanent micropillar array, the geometries of human ECs can be directed accordingly in a controllable manner. Owing to different cytoskeleton-mediated mechanotransduction, the migration and the focal adhesion/spreading of human ECs are respectively promoted in turn to facilitate the formation of a confluent EC monolayer, which appropriately match the dynamic ECM-mediated effects throughout the native endothelialization process. To the best of our knowledge, this is the first platform to enable stage-specific EC manipulation like the native ECM that induces endothelialization on demand. With the capability of stage-specific cell manipulation by transformable topographies, our bilayer platform offers the implications of not only promoting endothelialization, but also facilitating a broad range of applications such as wound healing and tissue engineering [35–37].

## RESULTS AND DISCUSSION

### NIR-triggered topographical conversion of the bilayer platform

**Figure 1.**
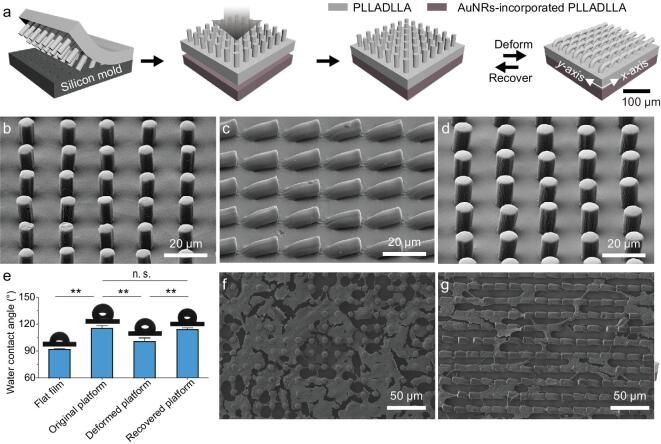
A bilayer platform based on a shape-memory polymer showing dynamic topographies. (a) The bilayer platform consists of one shape-memory layer with micropillar-array topography and one photothermal layer. These two layers are fabricated by replica molding and a casting method, respectively, and then bonded together. The topography of the bilayer platform can be altered based on the shape-memory effects of PLLADLLA. (b) The topography of the bilayer platform at its original shape shows periodic micropillars on the surface. (c) The topography of the bilayer platform at its temporary shape displays microgroove-array topography where the micropillars are bended in a head-to-tail manner with a certain orientation. (d) The topography of the bilayer platform at its recovered shape shows identical micropillar-array topography to that of the platform at the original shape. (e) Statistical analyses of the water-contact angles on the surfaces of platforms with different topographies. The platforms display similar wettabilities at their original and recovered statuses, which are statistically significantly different from those on the platform at the temporary state and the flat film. (f) The morphology of human umbilical vein endothelial cells (HUVECs) on the platform with micropillar-array topography, demonstrating stretching cell shapes supported by the micropillars of the platform. (g) The morphology of HUVECs on the platform with microgroove-array topography, demonstrating elongated cell shapes complying with the alignment of microgrooves on the surface of the platform.

Our bilayer platform consists of one shape-memory layer and one photothermal layer, where the topographical arrangement at the shape-memory layer of the platform is designed to be tuned by the heating of the photothermal layer upon NIR irradiation (Fig. 1a). The shape-memory layer of the bilayer platform was formed using a biodegradable shape-memory polymer, poly(L-lactide-*co*-D, L-lactide) (PLLADLLA), through a replica-molding method (Supplementary Fig. S1a). And the photothermal layer of the bilayer platform was formed by using PLLADLLA and a photothermal agent, gold nanorods (AuNRs), via a casting method. An as-prepared shape-memory layer and a photothermal layer possess outstanding mechanical properties (Supplementary Fig. S2a) that are superior to normal hydrogel-based actuating biomaterials [26,27], which will be compliant with human's soft tissues and thereby suitable for vascular-tissue-engineering applications [38]. These two layers were bonded through brushing a small amount of solvents on their interface, resulting in a bilayer platform with excellent structural integrity even under repeated folding/unfolding processes (Supplementary Movie. 1), as there is a negligible gap between the two layers (Supplementary Fig. S1b). Additionally, the resulting bilayer platform shows an ordered micropillar-array topography (Fig. 1b). The well-arranged periodic micropillar array is 7.5 μm in diameter, 20.0 μm center-to-center spacing and 15.0 μm in height in a square lattice, which can offer appropriate isotropic topographical guidance to promote the spreading of mammalian cells [39]. Upon the lodging of micropillars in a certain direction along the *x*-axis of the platform, the micropillars contact in a head-to-tail manner (Fig. 1c), resulting in a temporary microgroove-array topography for potentially offering anisotropic topographical cues to cells [40]. In addition to the flat-shaped platform, the original shape of the bilayer platform could be also processed to a three-dimensional tubular shape (Supplementary Fig. S3) due to the versatile processability of the materials, which would match the shapes of target vascular tissues for potential implantation applications [25]. Owing to the desirable shape-memory effects of PLLADLLA with a phase-transition temperature (*T_trans_*) of ∼53°C (Supplementary Fig. S2b), the platform can not only obtain a stable temporary microgroove-array topography at room temperature once micropillars bended directionally (Fig. 1c), but also completely recover to its permanent micropillar-array topography upon heating in an oven at 65°C for 15 min (Fig. 1d). The topographical recovery of the bilayer platform can be also reflected by their similar surface wettabilities (Fig. 1e). Specifically, the water-contact angles show no statistically significant difference between the original and the recovered platform, indicating their similar topographical arrangements. In contrast, the temporary platform and the control flat PLADLLA film that have different topographies show distinct water-contact angles on their surfaces due to the varying solid-area frictions.

**Figure 2.**
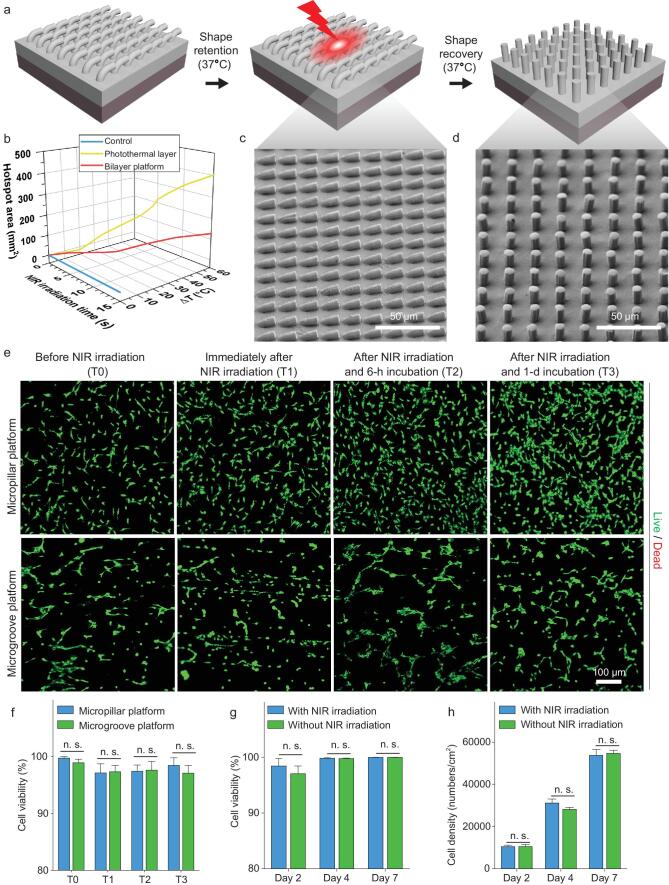
NIR-triggered topographical conversion of the bilayer platform. (a) A schematic illustration of the topographical alteration of the bilayer platform triggered by NIR irradiation. (b) Temperature increase (at center) and hotspot area (*T* > *T_trans_*) of neat PLLADLLA film (control), AuNRs-incorporated PLLADLLA film (photothermal layer) and bilayer platform irradiated by a NIR laser with a power of 5 W/cm^2^ at a distance of 3 cm over different time periods measured by infrared thermography, showing their distinct photothermal effects. (c) The topography of the temporary platform after immersion treatment at 37°C PBS for 24 h, demonstrating excellent retention of the temporary shape at the cell-culture condition. (d) The topography of the platform upon NIR irradiation, showing completely recovered micropillar array identical to its original state. (e) Representative fluorescence images showing the viability of HUVECs on the bilayer platform (with initially either micropillar topography or microgroove topography) before NIR irradiation (T0), immediately after 10-s NIR irradiation (T1), after NIR irradiation and the following 6-h incubation (T2), and after NIR irradiation and the following 1-d incubation (T3), demonstrating a negligible immediate influence of NIR irradiation on cell viability. (f) Statistical analyses of the viability of HUVECs on different platforms at specific time points (T0, T1, T2 and T3), showing that there is no statistically significant difference in the cell viability on the platforms with different original topographies. (g) Statistical analyses on the viability of HUVECs on the platforms with/without 10-s NIR irradiation within 7-d cell-culture periods, showing no statistically significant difference in the cell viability on different platforms. (h) Statistical analyses of the densities of HUVECs cultured on the bilayer platforms with/without 10-s NIR irradiation within 7-d cell-culture periods, showing that there is no statistically significant difference in the proliferation rates of HUVECs with/without NIR irradiation.

To trigger the topographical change of the bilayer platform for stage-specific cell manipulation, a remote-controlled way applicable at the thermostatic environment (37°C), rather than changing the overall environmental temperature, is preferred. NIR irradiation is visible for triggering the topographical alteration of our platform, as AuNRs with a high aspect ratio and obvious absorbance at the NIR region (Supplementary Fig. S2c) are incorporated into the bottom photothermal layer of the platform. The heat generated at the bottom photothermal layer upon NIR irradiation is expected to drive the phase transition of PLLADLLA and subsequently the topographical recovery of the upper shape-memory layer from its temporary microgroove-array topography to the permanent state (Fig. 2a). To examine the feasibility of the NIR irradiation to trigger the phase transition of PLLADLLA, the surface temperatures of the photothermal layer upon NIR irradiation were measured using an infrared thermometer.

Once the NIR laser was turned on, the surface temperature of the photothermal layer rapidly increased, exceeding the *T_trans_* of PLLADLLA within only 3 s (Fig. 2b). With extending exposure time, a higher temperature at the center and expanded areas of the hotspot (with temperature above the *T_trans_* of PLLADLLA) are present for the photothermal layer owing to more heat being generated and the thermal-diffusion effects (Fig. 2b and Supplementary Fig. S4). In comparison, there is a negligible increase in the temperature for the neat PLLADLLA film (control) upon NIR irradiation over time (Fig. 2b and Supplementary Fig. S4). As for the bilayer platform, the surface temperature of the shape-memory layer also obviously increases over different NIR-irradiation time periods (Supplementary Fig. S4), though the increasing rate is lower than that of the photothermal layer alone (Fig. 2b). After 10-s NIR irradiation, an obvious hotspot with an area over 100 mm^2^ and the temperature over the *T_trans_* of PLLADLLA is also formed on the surface of the bilayer platform (Fig. 2b and Supplementary Fig. S4). The results reveal that the heat generated from the bottom photothermal layer with excellent NIR-responsive photothermal effects can conduct to the upper shape-memory layer and will be sufficient to drive the phase transition of PLLADLLA *in situ*, which will be important for developing a remote and non-invasive-trigger way of controlling the topographical alterations in the future *in vitro* and *in vivo* implantation applications. Interestingly, when immersing the photothermal layer and the bilayer platform in water, the increases in their surface temperatures upon NIR irradiation are significantly lower than those in air (Supplementary Fig. S4), which is supposed to be attributed to the high specific heat capability and rapid thermal diffusion of water. It is therefore reasonable to infer that NIR irradiation can result in significant temperature gradients across the thickness of the bilayer platform to drive the phase transition of PLLADLLA yet

cause a slight temperature increase on its surface in water-rich environments (*in vitro* and *in vivo*), which will reduce the risk of cell damage.

Precisely controlled topographies in thermostatic physiological environments is the prerequisite of stage-specific cell manipulation by our bilayer platform. To verify the effectiveness of the NIR irradiation on altering the topographies of the bilayer platform in the physiological environment, the retention of the temporary topography and the recovery to the permanent topography of the bilayer platform in a cell-culture incubator (37°C, 5% CO_2_, humidity, 85 ∼ 95%) were evaluated. First, the topography of the temporary microgroove platform after 24-h phosphate buffer saline (PBS, pH = 7.4) immersion treatment in the cell-culture incubator was characterized, showing well-preserved microgroove topography (Fig. 2c) and therefore excellent shape-retention capabilities of the temporary platform in the physiological environment. Additionally, the temporary microgroove-array topography of the bilayer platform could completely recover to the permanent well-arranged micropillar-array topography upon 10-s NIR irradiation (Fig. 2d), while 5-s NIR irradiation only leads to partial topographical recovery of the bilayer platform with inclined micropillars (Supplementary Fig. S5). These results indicate that the topographies of the bilayer platforms have bi-stable states in the thermostatic physiological environments, which can be readily converted by 10-s NIR irradiation.

With the aim of stage-specific cell manipulation by our bilayer platform, the impacts of the heat generated by the bottom photothermal layer and the topographical conversion of the shape-memory layer upon NIR irradiation on the cell viability must be considered. To assess the influences of NIR irradiation on cell viability, a human-derived primary vascular endothelial cell, human umbilical vein endothelial cell (HUVEC), were seeded on the bilayer platform with different topographies and live/dead cytocompatibility assays were conducted. Regarding the HUVECs on the platforms with either micropillar or microgroove topography at different time points (before NIR irradiation, immediately after 10-s NIR irradiation, after NIR irradiation and the following 6-h incubation, and after NIR irradiation and the following 1-d incubation), there are few dead cells on these platforms with different topographies at each time point (Fig. 2e) and no statistically significant difference in the cell viability among the different groups (Fig. 2f). It can therefore be concluded that neither the topographical alteration nor the heat generated from the bottom photothermal layer upon NIR irradiation to drive the topographical conversion of the bilayer platform affects the viability of HUVECs. With an extending cell-culture time of up to 7 d, the viability and densities of HUVECs on the bilayer platform with/without NIR irradiation over time were also assessed. The results indicate that there is no statistically significant difference in both cell viability and proliferation for the HUVECs on the platforms with/without NIR irradiation over 7-d incubation (Fig. 2g and h, and Supplementary Fig. S6), further verifying the desirable biosafety by way of 10-s NIR irradiation to trigger the topographical conversion of the bilayer platform.

### Dynamic guidance on human EC geometries

**Figure 3.**
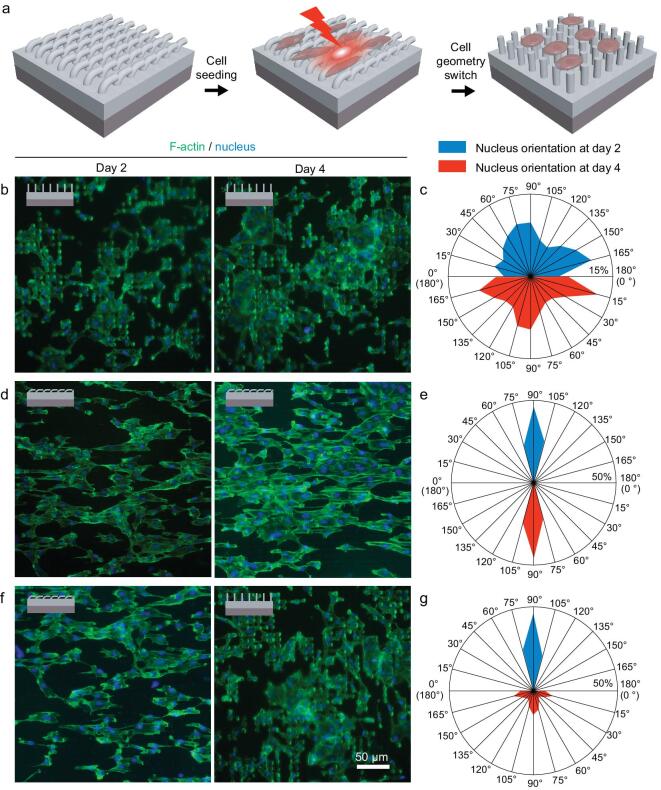
Modulating effects on the geometries of human ECs by the topographically dynamic platform. (a) A schematic illustration of the modulation of cell geometry by altering the topographies of the bilayer platform triggered by a NIR laser. (b) Representative fluorescence images of HUVECs on the platform with static micropillar-array topography on days 2 and 4 of incubation. (c) Analyses on the nucleus orientation of the HUVECs on days 2 and 4 of incubation on the platform with static micropillar-array topography (NIR-irradiation treatments performed on day 2 of incubation). (d) Representative fluorescence images of HUVECs on the platform with static microgroove-array topography on days 2 and 4 of incubation. (e) Analyses of the nucleus orientation of the HUVECs on days 2 and 4 of incubation on the platform with static microgroove-array topography (without NIR irradiation). (f) Representative fluorescence images of HUVECs on the platform with dynamic topography on days 2 and 4 of incubation. (g) Analyses of the nucleus orientation of the HUVECs on days 2 and 4 of incubation on the platform with dynamic topography (NIR-irradiation treatments performed on day 2 of incubation).

For the bilayer platform with dynamic topographies, the geometries of the cells were expected to be altered by the topographical conversion upon NIR irradiation (Fig. 3a). Although the NIR-triggered topographical conversion of the bilayer platform has been found to have no impact on the viability and proliferation of human ECs, its influence on the geometries of human ECs remains unknown. To unravel the modulating effects of the topographical conversion on cell geometry, the geometries of the HUVECs cultured on the platform with either permanent or temporary topography were first compared. It can be observed that HUVECs on the platform with a permanent micropillar-array topography exhibit isotropic shapes with expanded spreading areas (Fig. 1f), while anisotropic cell alignment along with the orientation of microgrooves are present for the HUVECs on the temporary platform (Fig. 1g). Based on the fact that different static topographies of the bilayer platforms direct distinct cell shapes, the influences of the topographical conversion of the bilayer platform upon 10-s NIR irradiation on the cytoskeleton organization and nucleus orientation of human ECs were then investigated. First, we find that HUVECs exhibit unaltered cytoskeleton arrangement and nucleus orientation on the platforms with static micropillar-array topographies before and after 10-s NIR irradiation (Fig. 3b and c), excluding the possibility of switching cell geometries by NIR irradiation alone yet without topographical conversion. Additionally, compared to the unaltered parallel aligned cytoskeleton organization and nucleus orientation of HUVECs on the platforms with static microgroove-array topographies after 2 and 4 d of culturing without NIR irradiation (Fig. 3d and e), obvious changes in the cytoskeleton organization and nucleus orientation from anisotropy to isotropy are present on days 2 and 4 of incubation for the HUVECs on the platform with initially the same topography yet with NIR irradiation (Fig. 3f and g). The topographical rearrangements of the platform triggered by 10-s NIR irradiation on day 2 is found to effectively reshape the cell morphology, actin filament (F-actin) alignment and nucleus orientation of HUVECs. After NIR irradiation and the following 2-d incubation, the HUVECs on the dynamic platform display similar geometries to those initially grown on the static micropillar platform. It can be therefore concluded that the unspontaneous geometrical rearrangements of human ECs can be triggered on demand by the NIR-controlled topographical conversion of the dynamic platform at the physiological temperature (37°C). The effective control over the geometries, particularly the polarization, of human ECs by the changing traction forces between the cells and the platform with varying topographies offers the basis to regulate specific EC functions both *in vitro* and *in vivo* via controllable mechanotransduction signaling, as well as the potential for stage-specific EC manipulation for inducing endothelialization resembling the native ECM-mediated effects.

### Stage-specific regulation of human EC functions for endothelialization

**Figure 4.**
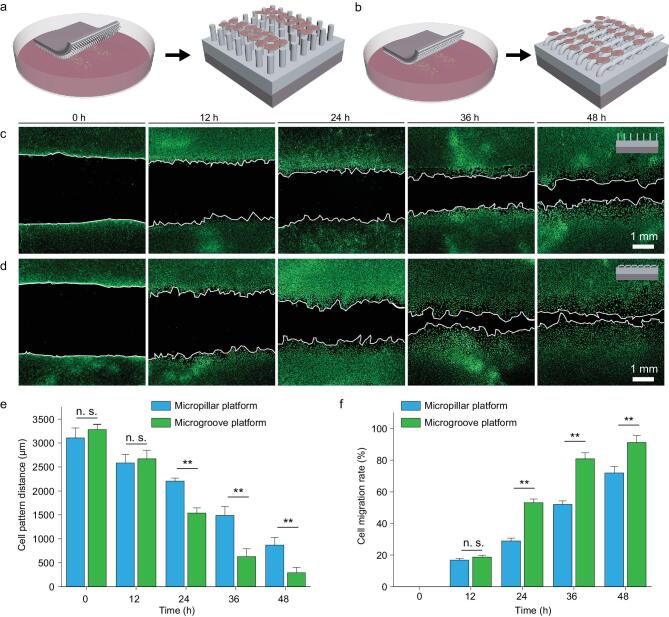
The migration of human ECs directed by the platforms with different original topographies. (a, b) Schematic illustrations of cell-migration assessments through a cells-on-chips method. The migration of HUVECs are assessed according to the changes in the spacing of cell patterns with predetermined distances, where the distances of cell patterns under the guidance of platforms with either original micropillar topography (a) or microgroove topography (b) over different cell-culture time periods are measured. (c) Representative fluorescence images of cell patterns guided by the micropillar platform over different cell-culture time periods (12, 24, 36 and 48 h). (d) Representative fluorescence images of cell patterns guided by the microgroove platform over different cell-culture time periods (12, 24, 36 and 48 h). (e) Statistical analyses on the average spacing of cell patterns over different cell-culture time periods guided by platforms with either permanent micropillar-array or temporary microgroove-array topography over different incubation time periods. (f) Migration rates of HUVECs guided by different platforms with either permanent micropillar-array or temporary microgroove-array topography over different incubation time periods.

To replicate the native ECM-mediated effects on directing EC functions throughout the endothelialization process, desirable biomaterials are expected to promote EC migration at the beginning stage and subsequently to assist cell proliferation/spreading to form a confluent EC monolayer at the later stage [3]. Our bilayer platform with initially temporary microgroove-array topography has been proven to effectively direct cell polarization, which is promising to promote cell migration along the direction of microgroove orientation by contact guidance [41,42]. To verify the inducing effects of the bilayer platform with initially temporary microgroove-array topography on EC migration, a cells-on-chips model was built according to the established method [43]. Specifically, dense HUVECs were confined in microchannels with a predetermined spacing of ∼3000 μm on the bottom of the Petri dishes by polydimethylsiloxane (PDMS) chips. Bilayer platforms with either microgroove or micropillar-array topographies were placed on the cell patterns after peeling off the PDMS chips. The migration of the HUVECs guided by different platforms with either micropillar (Fig. 4a) or microgroove-array topography (Fig. 4b) was then quantified according to the changes in the spacing between the cell patterns. It can be observed that there are few variances in cell migration between HUVECs guided by micropillar (Fig. 4c) and microgroove platforms (Fig. 4d) within the initial 12 h of culturing. However, with extending cell-culture time, distinctly different guiding effects of the platforms with different topographies on the migration of HUVECs have appeared. Indicated by different decreasing rates in the average

spacings between cell patterns guided by different platforms over time (Fig. 4e), the migration of HUVECs on the microgroove platform is found to be significantly faster than that on the micropillar platform (Fig. 4f). While clear barriers remain on the micropillar platform after 48-h incubation (Fig. 4c), the cell patterns get partially merged guided by the microgroove platform after the same cell-culture time (Fig. 4d). The results verify that the bilayer platform with initially temporary microgroove-array topography can effectively promote the collective migration of human ECs, which will meet the requirements of endothelialization at the early stage.

In addition to promoting EC migration in the beginning, the adhesion and spreading of human

**Figure 5.**
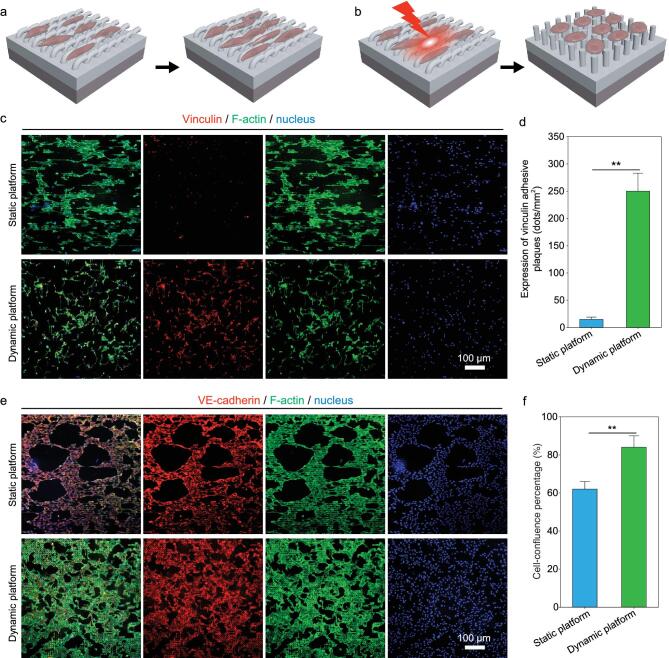
Focal adhesion and intercellular junction of human ECs on different platforms with either static topography or dynamic topography. Schematic illustrations of cell functions directed by either static platform (a) or dynamic platform (b). (c) Representative fluorescence images of HUVECs on different platforms on day 3 of culturing, showing similar cell density (indicated by DAPI-stained nucleus) yet distinctly different results in cytoskeleton organization and cell focal adhesion. (d) Statistical analyses on the expression of vinculin adhesive plaques for the HUVECs grown on different platforms on day 3 of culturing. (e) Representative fluorescence images of HUVECs on different platforms on day 7 of culturing, exhibiting both abundant expression of VE-cadherin and similar cell density (indicated by DAPI-stained nucleus), yet different conditions in cytoskeleton organization and cell-confluence rates. (f) Statistical analyses on the cell-confluence percentages of HUVECs grown on different platforms on day 7 of culturing, demonstrating a statistically significantly higher confluence rate of HUVECs on the platform with dynamic topography than that on the platform with static microgroove-array topography.

ECs are expected to be also enhanced by our bilayer platform with dynamic topographies to form a confluent EC monolayer at the later stage of endothelialization. For our bilayer platforms, their modulating effects on EC functions from facilitating cell migration to promoting cell adhesion/spreading are designed to be switched by NIR-triggered topographical conversion. To examine the effectiveness of altering the topographies on inducing different EC functions, focal adhesion and intercellular junctions of HUVECs cultured on the platforms with either static (Fig. 5a) or dynamic topographies (Fig. 5b) were investigated. Despite the similar densities of HUVECs on different platforms after 2 d of culturing, the geometries and focal adhesion status are distinctly different for the cells cultured on the platforms with/without NIR irradiation (Fig. 5c). HUVECs on the static platform without NIR irradiation exhibit anisotropic cell morphology and F-actin alignment along with the orientation of microgrooves yet limited expression of vinculin adhesive plagues (Fig. 5c). In comparison, HUVECs on the dynamic platform with NIR-triggered transformable topographies show clear changes in cytoskeleton organization before and after NIR irradiation, which is shifted from initially anisotropic cytoskeleton organization with elongated F-actin arrangements (Supplementary Fig. S7) to subsequently isotropic cytoskeleton organization with randomly oriented and well-stretched F-actin arrangements upon the topographical recovery of the platform (Fig. 5c). It is also important to note that remarkably enhanced focal adhesion of HUVECs is achieved on the dynamic platform after NIR irradiation, as the recovered micropillar-array topography of the dynamic platform with a high surface-area-to-volume ratio favors the formation of abundant cell anchoring between the materials and cells [44], which is also indicated by the statistically significant higher expression of vinculin adhesive plaques on the dynamic platform than that on the static platform (Fig. 5d). These results verify that the focal adhesion of human ECs can be effectively enhanced by the dynamic platform after NIR-triggered topographical conversion, which will be meaningful for the firm adhesion of human ECs on the materials to resist the shear stress of blood flow for the initialization of a confluent EC monolayer [45].

To further evaluate the effects of platforms with different topographies on the formation of a confluent EC monolayer *in vitro*, the intercellular junctions among ECs on different platforms were investigated within extending cell-culture time for up to 7 d. After 7-d incubation, the nucleus, F-actin and an important cytoskeleton protein indicating intercellular junctions, VE-cadherin, of HUVECs on either static or dynamic platforms were labeled and observed. On the static platform without NIR-triggered topographical conversion, HUVECs after 7 d of culturing still exhibit significantly anisotropic cell geometries like the condition on day 2 of incubation (Fig. 5c and e), demonstrating excellent shape-retention performance of the temporary microgroove-array topography for the platform in the cell-culture condition and its durable effects on directing cell geometries. However, despite abundant intercellular junctions indicated by the high-level expression of VE-cadherin (Fig. 5e), the HUVECs on the static platform still show a relatively low cell-confluence percentage of ∼60% (Fig. 5f). Restricted by narrow and contractile cell shapes on the platforms with unaltered microgroove-array topography, many large voids remain on the EC monolayer even after 7-d cell culture, corresponding to the pathological condition of the disruptive vascular barrier functions [46]. In comparison, the HUVECs on the dynamic platform with NIR-triggered topographical conversion form an EC monolayer with abundant intercellular junctions, fewer voids and higher integrity, with a cell-confluence percentage of >80% after 7-d cell culture (Fig. 5e and f). While the static platform with the topography to initially promote EC migration would delay the endothelialization process at the later stage if the topography was not converted, abundant intercellular junctions and statistically significantly higher cell-confluence percentages of the HUVECs on the dynamic platform both demonstrate the effectiveness of altering the topographies on promoting the formation of a confluent EC monolayer at the later stage of endothelialization [47], as well as facilitating rapid endothelialization in a step-by-step manner.

## CONCLUSION

In summary, we establish a topographically dynamic bilayer platform with the capabilities of stage-specific EC manipulation resembling the dynamic native ECM-mediated effects. The topographical conversion of the platform from anisotropy to isotropy enabled by NIR irradiation at the thermostatic physiological condition (37°C) can lead to differentiated guidance on cell geometry and subsequently stage-specific enhancements of the migration and the adhesion/spreading of human ECs in turn, yet exhibits negligible damage on cell viability. With the capabilities of inducing specific EC functions in a well-defined sequential manner, our topographically dynamic platform effectively promotes rapid endothelialization, which holds great promise in vascular-tissue engineering. Notably, stage-specific cell manipulation (i.e. initially promoting cell migration/recruitment and subsequently enhancing cell adhesion/spreading) is not only required in the endothelialization process, but also important in wound healing and the regeneration of many human tissues [48–50]. It can be therefore envisioned that our platform enabling stage-specific cell manipulation will inspire the construction of advanced biomimetic dynamic cell microenvironments for meeting the complicated requirements of different tissue-repair applications.

## METHODS

### Bilayer-platform preparation

Our bilayer platform consists of a shape-memory layer and a photothermal layer, which are separately fabricated by different methods. The shape-memory layer with micropillar-array topography is made of a biodegradable shape-memory polymer, PLLADLLA (Jinan Daigang Ltd), which was prepared by replication from a silicon mold with periodic microhole array (7.5 μm in diameter, 20.0 μm center-to-center spacing and 15.0 μm in depth in a square lattice) using the established replica-molding method [51]. In brief, the master silicon mold was prepared through photolithography with SU8–3010 photoresists (MicroChemicals, GmbH) and a following inductively coupled plasma (ICP) etching process using an ICP facility (DSE200, Northern Micro-electronics Co., Ltd). Prior to pouring a 10-mL PLLADLLA solution (8 w/v%, dissolved in CH_2_Cl_2_) into the silicon mold, an as-prepared silicon mold was first treated by oxygen plasma (Gatan Solarus Model 950) at the parameters of the plasma power of 65 W, pressure of 70 mTorr and O_2_ flow of 40 sccm for 2 min to increase the density of –OH groups on the surface and subsequently treated by fluoroalkylsilane at room temperature in a vacuum oven for 15 min. The PLLADLLA film with micropillar-array topography and a thickness of ∼150 μm was formed after complete removal of the solvents via drying in air (25°C) overnight, in a vacuum oven at 35°C for 3 d and then at 25°C for 3 d. AuNRs-incorporated PLLADLLA film was prepared as the photothermal layer of the bilayer platform through a casting method referring to our previous study [52]. First, AuNRs were synthesized using an established seed-mediated growth method [53]. The suspension of AuNRs was then centrifuged at 10 000 rpm for 15 min to be condensed to a final concentration of 4 mM. The condensed suspension of AuNRs was centrifuged again, followed by the removal of supernatant and the dropping of the remaining deposits into a 5-mL PLLADLLA solution (8 w/v%, dissolved in trifluoroethanol) to form a mixture solution under vigorous stirring. The AuNRs-incorporated PLLADLLA film with a thickness of ∼150 μm was formed through the casting of the mixture solution in a 2-inch glass Petri dish, which was kept at room temperature overnight and then in a vacuum oven (25°C) for 3 d to completely remove the residual solvents. After the preparation of the PLLADLLA film with micropillar-array topography and the AuNRs-incorporated PLLADLLA film, these two films were pasted together with the assistance of a small amount of CH_2_Cl_2_ brushed on their interfaces, resulting in a bilayer platform with a total thickness of ∼350 μm and a permanent micropillar-array topography. For constructing the temporary microgroove-array topography of the bilayer platform, the platform was warmed up in an oven at 65°C for 15 min, scraped using a polished glass slide to press down the micropillars along the *x*-axis of the platform and then placed in a refrigerator (4°C) for 30 min to fix its temporary shape. To investigate the processability of the bilayer platform, the bilayer platform with a permanent tubular shape and an inner diameter of ∼3 mm was prepared via the established template-assisted method [25,54], where the layer with micropillar-array topography was located on the lumen of the bilayer tube. The shape-memory performances of resulting tubularly shaped platform were examined at the reshaping process to obtain a temporary flat shape with microgroove-array topography and the shape-recovery process by heating via the similar above-described procedures.

### Material characterizations

The *T_trans_* of PLLADLLA was determined from the differential scanning calorimetry (DSC) curve of the heat flow with respect to the temperature, which was obtained at the second heating process according to a predetermined heating–cooling program between −50 and 150°C using a DSC testing machine (TA Instruments, Q20). The structure and the UV-visible absorption spectra of as-synthesized AuNRs were characterized by a field-emission transmission electron microscope (JEM-2100, JEOL Ltd) and a spectrophotometer (UV 2611, Shimadzu), respectively. The microstructure and morphology of the bilayer platform were examined using a field-emission scanning electron microscope (SEM, Sigma, Carl Zeiss). The SEM images viewed at the cross-section, the 45° dip angle and the top of gold-sputtering samples were recorded. The macroscopic shapes of as-prepared samples were recorded using a digital camera (EOS 7D Mark II, Canon). Tensile tests were performed for the individual shape-memory layer and the photothermal layer of the bilayer platform separately using a universal testing machine (AG-X Plus 100 N, Shimadzu) under the American Society for Testing Materials test standard (ASTM C1557). Samples for tensile tests were cut into stripes with a width of ∼1.00 cm and a length of ∼2.50 cm, whose accurate dimensions (width, length and thickness) were measured by a digital caliper. The tensile tests were conducted at a loading of 100 N and a constant tensile rate of 2 mm/min until the breaking-down of the stripes. The strain–stress curves were plotted and the mechanical properties (Young's modulus, ultimate tensile stress and fracture strain) of the shape-memory layer and the photothermal layer were statistically analysed (*N* = 6). To evaluate the shape-memory performance of the as-prepared bilayer platform, the recovered microstructure and morphology of the bilayer platform were characterized by SEM after settling the temporary platform in an oven (65°C) for 15 min. The static water-contact angles on the surfaces of platforms with original topography, deformed temporary topography and recovered topography were measured on a contact-angle-measurement platform (DSA25, Kruss, Germany), where a flat PLLADLLA film was set as control. In the static water-contact-angle measurement, a 5.0-μL water droplet was placed onto a square-shaped sample. The stabilized shape of the water droplet was recorded. And the static water-contact angle of each platform was calculated accordingly and statistically analysed (*N* = 6). To study the preservation of the temporary microgroove topography of the platform, the microstructure and morphology of the temporary platform after the PBS immersion treatment at 37°C for 24 h were characterized by SEM. To investigate the NIR-triggered photothermal effects of the photothermal layer and the bilayer platform, the infrared thermal images of the AuNRs-incorporated PLLADLLA film and the bilayer platform upon NIR irradiation (5, 10 or 15 s) both in air and in water at room temperature using a NIR laser (5 W/cm^2^, Nanjing Laichuang Laser Technology Co., Ltd) at a distance of 3.00 cm were captured using an infrared thermometer (R300SR, NEC). The neat PLADLLA formed by a casting method was set as control. The microstructure and morphology of the temporary platform upon NIR irradiation (5 or 10 s) were characterized by SEM for assessing the NIR-induced topographical alteration for the bilayer platform.

### Cell culture

Primary HUVECs were obtained from ScienCell, USA. HUVECs were cultured in a recommended endothelial culture medium (ScienCell) with supplemented 5 v/v% fetal bovine serum (FBS, ScienCell), 1 v/v% penicillin/streptomycin (P/S, ScienCell) and 1 v/v% endothelial cell-growth supplement (ESGS, ScienCell) in a 37°C and 5% CO_2_ incubator with a humidified atmosphere. The culture medium was refreshed every 2 d. With a cell confluence of ∼80%, HUVECs were trypsinized by a 0.025 w/v% trypsin/EDTA solution (Gibco) and a neutralizer solution (Gibco) to prepare a cell suspension for cell seeding. HUVECs at passages 2 and 3 were used in this study. Materials for cell seeding were sterilized via the immersion treatment in a 75 v/v% ethanol solution overnight and then rinsed three times with PBS (Sigma-Aldrich).

### Cell-viability assay

The viability of HUVECs cultured on different platforms over different cell-culture time periods was assessed through live/dead cell staining using a LIVE/DEAD Viability/Cytotoxicity Kit (Invitrogen) according to the manufacture's protocol. Live cells and dead cells were respectively labeled by Calcein AM (green color) and ethidium homodimer-1 (red color), which can be observed on an inverted fluorescence microscope (Ti-U, Nikon). In this experiment, HUVECs were seeded on the platforms with either permanent micropillar-array or temporary microgroove-array topographies at a cell density of 1 × 10^4^ cells/cm^2^ and then incubated for 1 d for attachment on the platforms. NIR irradiation was then performed. HUVECs on the platforms before NIR irradiation (T0), immediately after 10-s NIR irradiation (T1), after NIR irradiation and the following 6-h incubation (T2), and after NIR irradiation and the following 1-d incubation (T3) were stained and observed, aiming to evaluate the immediate influence of NIR irradiation on cell viability. HUVECs on the platforms with/without NIR irradiation were then incubated for additional 2 and 6 d, which were also stained by Calcein AM and ethidium homodimer-1 and then observed on the inverted fluorescence microscope for investigating the chronic influence of NIR irradiation on cell viability. Statistical analyses (*N* = 3) were conducted for comparing the viability and density of HUVECs on the platforms with different topographies and with/without NIR irradiation over different cell-culture time periods through the analyses on the images using the ImageJ software (National Institutes of Health, USA).

### Cell-geometry evaluation

To investigate the influence of different topographies of the platform on cell geometry, HUVECs cultured on the platforms with either temporary microgroove-array topography or permanent micropillar-array topography after 1-d incubation for cell attachment were characterized by SEM. In brief, HUVECs on different platforms after 1-d cell culture were rinsed three times with PBS, fixed with a 3 v/v% glutaraldehyde solution for 3 h, treated by ethanol solutions with gradient ethanol concentrations (30, 50, 75, 80, 95 and 100 v/v%), dried using a critical-point dryer (Autosamdri-815B, bbe Moldaenke) and then coated with a thin layer of gold by sputtering for SEM characterizations. To investigate the influence of topographical alteration on cell geometry, HUVECs cultured on the bilayer platform with dynamic topographies were stained by Alexa Fluor 488 Phalloidin (A12379, Invitrogen) and 4′,6-diamidino-2-phenylindole (DAPI, D1306, Invitrogen) for respectively labeling the actin filament (F-actin, green color) and the nucleus (blue color), which were then visualized using the inverted fluorescence microscope. HUVECs were stained before and after NIR irradiation respectively on days 2 and 4 of culturing, while NIR irradiation was performed on day 2. The F-actin and nucleus of HUVECs on the platforms with static topographies (with either static micropillar-array topography or static microgroove-array topography) were also stained at the same time points of incubation and then visualized. The nucleus orientations of the HUVECs on the platforms on days 2 and 4 of incubation were also assessed through the measurements of the angles of the long axis of the nucleus with respect to the *y*-axis of the platform (*N* > 100), which were analysed from the fluorescence microscopic images using the ImageJ software.

### Cell-migration assessment

The migration of HUVECs guided by platforms with different topographies was assessed using the established cell-on-a-chip method [43]. To be specific, PDMS (Dow Corning Holding Co., Ltd) chips with two microchannels of lengths of 20 mm, heights of 2 mm, widths of 2 mm and a spacing of 3 mm were first fabricated by replica molding from a micromachined Lucite master mold. As-prepared PDMS chips were bonded onto the bottom of glass Petri dishes (Corning) after the oxygen-plasma treatment on their contact surfaces. Suspensions of HUVECs with a cell density of 1 × 10^4^ cells/mL were then filled in the microchannels of the PDMS chip. After 1-d incubation, the PDMS chip was peeled off, where two patterns of dense HUVECs with a spacing of ∼3.0 mm were left on the bottom of the Petri dish. A sterilized platform with either permanent micropillar-array topography or temporary microgroove-array topography was placed above the cell pattern, where the microgroove orientation of the temporary platform was orthogonal to the long axis of the cell pattern. At specific time points of culturing (0, 12, 24, 36 and 48 h), HUVECs in the cell patterns guided by platforms with different topographies were stained by Calcein AM and visualized on the inverted fluorescence microscope. Cell migration directed by the topographies of different platforms was evaluated through the statistical analyses on the average spacings of the cell patterns over different cell-culture time periods from the measurements of cell-pattern distances at seven random positions from three separate experiments. The cell-migration rate (*V_i_*) was calculated according to Equation (1):

(1) \begin{equation*}{V_i}\ = {D_i}/{D_0}\ \times \ 100\% , \end{equation*}

where *D_0_* represents the average spacing between the initial cell patterns and *D_i_* represents the average spacing between the cell patterns guided by different platforms at specific time points.

### *In vitro* endothelialization evaluation

HUVECs were seeded on sterilized platforms with temporary microgroove topographies with a cell-seeding density of 1 × 10^4^ cells/cm^2^. While HUVECs in the control were cultured on the platforms for up to 7 d without any further treatment, NIR irradiation was applied in the experimental group after 1-d incubation for cell attachment. On day 3 of incubation, the expression of vinculin for the HUVECs in the control and on the platforms upon NIR irradiation were evaluated by immunochemical staining. In brief, HUVECs on different platforms after 2-d cell culture were fixed with a 4 v/v% paraformaldehyde PBS solution for 10 min, permeabilized with a 0.5 v/v% Triton-X-100 solution for 10 min, blocked with a block solution [PBS with supplemented 1 w/v% bovine serum albumin (BSA, Sigma-Aldrich), 0.1 v/v% Tween 20 (Sigma-Aldrich) and 0.3 M glycine (Sigma-Aldrich)] for 1 h and then incubated with a working solution containing 1 w/v% BSA, Alexa Fluor 488 Phalloidin at a dilution of 1/100 and a recombinant anti-vinculin antibody (ab129002, Abcam) at a dilution of 1/50 at 4°C overnight, followed by the incubation of a working solution containing 1 w/v% BSA, 50 μg/mL DAPI and a Alexa Fluor 568 goat anti-rabbit secondary antibody (ab175471, Abcam) at a dilution of 1/200 at room temperature for 1 h. The stained samples were visualized on the inverted fluorescence microscope. The expression levels of vinculin for the HUVECs cultured on different platforms were quantified through the statistical analyses (*N* = 6) on the number of vinculin adhesive plaques per 1 mm^2^, which were calculated according to the fluorescence microscopic images with the assistance of the ImageJ software. On day 7 of incubation, HUVECs cultured on different platforms were treated and stained through the similar above-described procedures for labeling the nucleus, F-actin and VE-cadherin, respectively. While the nucleus and F-actin were stained by DAPI and Alexa Fluor 488 Phalloidin, respectively, VE-cadherin was stained by using a rabbit polyclonal anti-VE-cadherin antibody (ab33168, Abcam) and then the Alexa Fluor 568 goat anti-rabbit secondary antibody according to the manufacture's protocol. The confluent levels of EC monolayers on different platforms were statistically analysed according to the fluorescence microscopic images from three separate experiments.

### Statistical analysis

All quantitative results were analysed on Prism 6 software and presented as means ± standard deviation unless otherwise specified. Data were statistically analysed by analysis of variance (ANOVA) and the Tukey test. A value of *P* < 0.05 (*) or *P* < 0.01(**) was considered as statistically significant.

## Supplementary Material

nwz188_Supplemental_Files
