# Corrigendum to A stage-specific cell-manipulation platform for inducing endothelialization on demand

**DOI:** 10.1093/nsr/nwaa302

**Published:** 2021-03-18

**Authors:** Qilong Zhao, Juan Wang, Yunlong Wang, Huanqing Cui, Xuemin Du

In the Fig. 3 of  ‘A stage-specific cell-manipulation platform for inducing endothelialization on demand’ (*National Science Review*, Volume 7, Issue 3, 2020, Pages 629–643, doi: 10.1093/nsr/nwz188), the data for the representative fluorescence image of HUVECs on the platform with static microgroove-array topography on day 2 of incubation was incorrectly provided (Fig. 3d, left). The corrected version of Fig. 3 is presented below.

**Figure 3.**
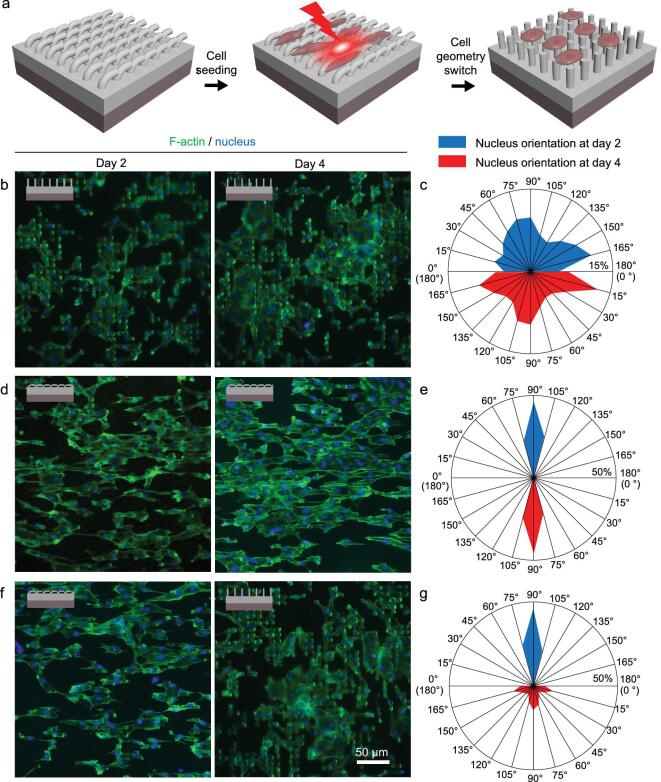
**Figure 3.** Modulating effects on the geometries of human ECs by the topographically dynamic platform. (a) A schematic illustration of the modulation of cell geometry by altering the topographies of the bilayer platform triggered by a NIR laser. (b) Representative fluorescence images of HUVECs on the platform with static micropillar-array topography on days 2 and 4 of incubation. (c) Analyses on the nucleus orientation of the HUVECs on days 2 and 4 of incubation on the platform with static micropillar-array topography (NIR-irradiation treatments performed on day 2 of incubation). (d) Representative fluorescence images of HUVECs on the platform with static microgroove-array topography on days 2 and 4 of incubation. (e) Analyses of the nucleus orientation of the HUVECs on days 2 and 4 of incubation on the platform with static microgroove-array topography (without NIR irradiation). (f) Representative fluorescence images of HUVECs on the platform with dynamic topography on days 2 and 4 of incubation. (g) Analyses of the nucleus orientation of the HUVECs on days 2 and 4 of incubation on the platform with dynamic topography (NIR-irradiation treatments performed on day 2 of incubation).

